# Pre-operative prediction of BCR-free survival with mRNA variables in prostate cancer

**DOI:** 10.1371/journal.pone.0311162

**Published:** 2024-10-01

**Authors:** Autumn O’Donnell, Michael Cronin, Shirin Moghaddam, Eric Wolsztynski

**Affiliations:** 1 School of Mathematical Sciences, Western Gateway Building, University College Cork, Cork, Ireland; 2 Department of Mathematics and Statistics (MACSI), University of Limerick, Limerick, Ireland; 3 Insight SFI Centre for Data Analytics, Dublin, Ireland; 4 Limerick Digital Cancer Research Centre (LDCRC), University of Limerick, Limerick, Ireland; State University of New York at Oswego, UNITED STATES OF AMERICA

## Abstract

Technological innovation yielded opportunities to obtain mRNA expression data for prostate cancer (PCa) patients even prior to biopsy, which can be used in a precision medicine approach to treatment decision-making. This can apply in particular to predict the risk of, and time to biochemical recurrence (BCR). Most mRNA-based models currently proposed to this end are designed for risk classification and post-operative prediction. Effective pre-operative prediction would facilitate early treatment decision-making, in particular by indicating more appropriate therapeutic pathways for patient profiles who would likely not benefit from a systematic prostatectomy regime. The aim of this study is to investigate the possibility to leverage mRNA information pre-operatively for BCR-free survival prediction. To do this, we considered time-to-event machine learning (ML) methodologies, rather than classification models at a specific survival horizon. We retrospectively analysed a cohort of 135 patients with clinical follow-up data and mRNA information comprising over 26,000 features (data accessible at NCBI GEO database, accession GSE21032). The performance of ML models including random survival forest, boosted and regularised Cox models were assessed, in terms of model discrimination, calibration, and predictive accuracy for overall, 3-year and 5-year survival, aligning with common clinical endpoints. Results showed that the inclusion of mRNA information could yield a gain in performance for pre-operative BCR prediction. ML-based time-to-event models significantly outperformed reference nomograms that used only routine clinical information with respect to all metrics considered. We believe this is the first study proposing pre-operative transcriptomics models for BCR prediction in PCa. External validation of these findings, including confirmation of the mRNA variables identified as potential key predictors in this study, could pave the way for pre-operative precision nomograms to facilitate timely personalised clinical decision-making.

## Introduction

Although radical prostatectomy (RP) has been the primary treatment for Prostate Cancer (PCa) for the last forty years, post-operative recurrence remains high [1]. This event is determined by a rise in Prostate Specific Antigen (PSA) and is termed a biochemical recurrence (BCR). BCR occurs in 20-40% of patients following RP [2]. Thus, determining factors that influence the time-to-BCR is critical for treatment decision-making. In particular, the ability to predict treatment failure pre-operatively could facilitate earlier decision-making regarding primary treatments and adjuvant therapies [3], including as to whether to recommend RP to patients at higher risk of recurrence. Recent technological improvements have created the opportunity to obtain patient genetic data routinely, which could be used in a precision medicine approach to inform therapeutic decisions. Identifying clinically useful variables with a strong potential for prediction of BCR is the main objective of this study. Specifically, we aim to examine the potential for inclusion of messenger ribonucleic acid (mRNA) transcriptomic information. In doing so, this work also evaluates the potential of machine learning (ML) methodologies over traditional methods and state-of-the-art nomograms. To date, most mRNA-based models proposed to this end are designed for risk classification and post-operative prediction. Effective pre-operative prediction would facilitate early treatment decision-making, in particular by indicating more appropriate therapeutic pathways for patient profiles who would likely not benefit from a systematic prostatectomy regime.

The Kattan nomogram for time-to-BCR prediction has been widely validated and is still commonly used for baseline comparison due to its high AUC (0.80) when initially assessed [4]. The Memorial Sloan Kettering Cancer Center (MSKCC) web-based nomogram is an update on the Kattan nomogram, with the most recent version dated April 2021 yielding a C-index of 0.80 on validation of a single-centred site [5]. While these nomograms have good performance metrics, they are subject to limitations. As the Kattan model was created in 1998, some criteria in its design are outdated. For instance, the tumour grading protocol used for Gleason scoring has changed so that the lowest score (originally 5) now assigned is 6 [6]. The PSA level deemed to indicate BCR has also changed from ≥ 0.4 ng/mL to having two PSA measurements ≥ 0.2 ng/mL [7].

Both models utilise routine preoperative clinical variables only, despite advances being made in this field to generate and integrate high-throughput patient-specific information. To date, predictive models that use mRNA information have been considered mainly in post-operative settings for the prediction of BCR [8, 9] and metastasis [10]. As the method currently implemented for mRNA extraction relies on tumour samples, genetic information generally only becomes available post-operatively. However, it is now possible to obtain this information from pre-operative needle biopsy, or more recent techniques again such as liquid biopsy, which gives rise to potential inclusion of mRNA information earlier in the patient care timeline. A number of studies have shown that the genetic profile at biopsy is representative of the entire tumour [11–13]. With this in mind, we assume that pre-operative mRNA information aligns strongly with that obtained during RP used in our models. In this study, we used mRNA data acquired post-operatively as a proxy for pre-operative modelling, together with pre-operative routine clinical variables, to produce the first pre-operative model for prediction of time-to-BCR. Other pre-operative models found in the literature are classifiers designed for binary prediction of recurrence or metastasis [14, 15], rather than time-to-event prediction models. Moreover, the selection of relevant mRNA biomarkers is performed here from within a set of over 26,000 mRNA variables, independently of previous findings, unlike in other (post-operative) mRNA-based models [8] that used predefined sets of variables. As such, the proposed study also proposes a benchmark of ML methodologies against conventional clinical modelling strategies for pre-operative time-to-BCR prediction.

## Materials and methods

### Data

The dataset was developed in a study by Taylor *et al* [16] containing 232 patients, of whom 198 had BCR-free survival time, along with censoring status, routine pre-operative clinical variables (namely patient age, PSA at diagnosis, Gleason score, ethnicity and clinical stage), and over 26,000 mRNA transcriptome variables. The data was accessed on the 1st of May 2021 and was fully anonymised at source (data accessible at NCBI GEO database, accession GSE21032). The mRNA data was obtained from surgical specimens during RP. Six patients with missing information were removed from the dataset, imputation not being feasible due to the large amount of missing information for each case, the bias introduced by imputation would likely outweigh that of selection bias. A further two patients had identical values for all variables except metastatic status and were thus both removed. Gleason score of 5 (n = 2), and Gleason score of 6 (n = 100) were combined to align with current grading protocols [6]. Of the 190 remaining patients, 135 had mRNA information. Our analyses were thus carried out on this sub-cohort.

### Models

In this study, follow-up times varied between patients, and the sole event of interest was BCR. Censored patients were included for analysis up until censoring occurred. Let the right-censored survival information recorded for the *N* patients in this cohort be denoted by

$${\left(T,\delta \right)}_{i},i=1,\cdots ,N$$

where *T* ≥ 0 and *δ* ∈ {0, 1} are respectively BCR-free survival time and censoring status (*δ* = 0 indicating the patient observation was censored at time *T*). Letting *T*^0^ be the true BCR-free survival time, and *τ*^0^ the true censoring time, then *T* = *min*(*T*^0^, *τ*^0^). Let also *m* ≤ *N* be the number of BCR events in this cohort, at distinct times

$$t_{1}<t_{2}<\cdots <t_{m},$$

and *d*_*j*_ BCR events recorded at time *t*_*j*_, *j* = 1, …, *m*. Given an *N*×*P* set **X** = (*X*_1_, …, *X*_*P*_) of *P* variables recorded for each patient, four distinct modelling strategies with the capacity to adjust for censoring were applied to the data for survival analysis of the time to BCR (${\left\{{\left(T,\delta ,X\right)}_{i}\right\}}_{i=1}^{N}$). From this analysis it was possible to derive the survival curve *S*_*i*_(*t*) = *Pr*(*T*_*i*_ > *t*) and associated survival characteristics of each patient. As several subsets of **X** were considered in our analyses, hereafter *P* loosely denotes the number of features in the relevant subset. One of the methods we considered for survival analysis was the conventional Cox proportional hazard model [17]. A limitation of this model is its inapplicability to wide datasets, where the number of predictors *P* far exceeds the number of observations *N*. To overcome this limitation, a forward stepwise selection (FS) was used, selecting only the most important features. As ML methodologies are widely recognised as being better suited for this type of high dimensionality, LASSO Cox [18] and boosted Cox [19] models were considered as alternatives to FS. The fourth strategy considered was the random survival forest (RSF), which consists of bagging survival trees [20]. The chosen ML models were selected for their prevalence in the literature and their shown improved performance in previous studies in cancer [21–23]. Different models were produced for each of these techniques based on different variable subsets, with sixteen models created. Firstly, Cox, RSF, LASSO Cox and boosted Cox models were developed using routine pre-operative clinical variables only, and compared to the current MSK nomogram for reference. The other twelve models were built using combined clinical and mRNA variable subsets obtained following either (unsupervised) correlation pre-filtering, or (supervised) univariate Cox feature selection, or both. The correlation filter was applied to the entire dataset, using an absolute cut-off value of 0.6 on Pearson’s correlation [24], a threshold deemed optimal in previous studies on similar data [25]. Correlation was used as a pre-filtering technique as it not only allows for dimensionality reduction but provides an unsupervised method to reduce multicollinearity in the data [26]. Univariate Cox feature selection of mRNA variables was performed on each of the resampled training sets, and the 50 variables with the best log-rank scores were retained in line with methods used by Beer *et al* on their assessment of gene expressions and the prediction of lung cancer [27]. Feature selection based on univariate Cox proportional hazard analyses is a supervised technique commonly used in clinical applications, that consists in electing standalone predictors for multivariate modelling [28]. FS was performed on all Cox models i.e. with or without feature pre-filtering/selection, allowing for a maximum of 10 variables to ensure a reasonable *N*/*P* ratio. Recursive feature elimination was performed on all RSF models. The hyperparameters of the RSF and boosted Cox models were kept to conservative settings [29, 30]. Thus, the RSF models were obtained using 500 trees, considering $\sqrt{P}$ variables at each split and with a minimum size of 15 for the terminal nodes, and boosted Cox models were built using 100 boosting iterations and with a fixed shrinkage parameter of 0.1. The regularisation parameter (λ) was selected using nested cross-validation for the LASSO Cox models [31], using a final value for λ corresponding to one standard deviation of the cross-validated error, as a standard approach to achieve parsimony [32].

### Evaluation framework

The 16 models described above were evaluated with respect to model discrimination, calibration, predictive performance and feature selection stability, via bootstrapping (using 100 iterations, i.e. where data was repeatedly sampled with replacement to create a dataset for training of equal size to the full dataset and the unselected samples, or out-of-bag (OOB) points, were used for testing). All analyses were carried out using the statistical software environment R [33] version 4.0.2.

#### Discrimination

In this study, discrimination refers to a model’s ability to correctly rank patients relative to each other based on their predicted time-to-BCR. A bootstrap-corrected concordance index (C-index), defined as the proportion of all feasible pairs of patients whose predicted and observed outcomes are concordant [34], and associated confidence interval (CI), were derived for each model for unbiased estimation of external predictive discrimination.

$$C=\hat{P}\left(\hat{S_{i}}\left(t\right)>\hat{S_{j}}\left(t\right)|T_{i}>T_{j}\right)$$

Bootstrap-corrected C-indices take into consideration the difference between the bootstrap sample models performance and the performance of the model built using the full dataset penalising for overfitting.

#### Calibration

Two forms of calibration analyses were used to assess model bias [35]. The first method is the more commonly implemented specific time-point method which aims to assess the conformity of a model to a binary outcome and is described in more detail in Harrell et al. [35]. The second evaluates the models conformity of a continuous model response typical of survival analysis. Both have been derived based on the OOB survival curve estimates ${\left\{{\hat{S}}_{i}\left(t\right)\right\}}_{i=1}^{N}$ yielded by each model for all *N* patients. Since the MSK nomogram is specifically calibrated to 5-year survival, calibration curves at the 5-year horizon were obtained with respect to quintiles $\left\{{\tilde{p}}_{1},\ldots ,{\tilde{p}}_{5}\right\}$ of the sample of values ${\left\{{\hat{p}}_{i}^{5}\right\}}_{i=1}^{N}$, where ${\hat{p}}_{i}^{5}={\hat{S}}_{i}\left(5\;\text{years}\right)=\hat{Pr}\left(T_{i}>5\;\text{years}\right)$ denotes the probability for patient *i* to survive at least 5 years. Points on the calibration curves have coordinates $({\bar{S}}_{q}^{5},{\hat{S}}_{KM,q}^{5}),q=1,\ldots ,5$, where ${\bar{S}}_{q}^{5}$ denotes the sample mean survival likelihood

$${\bar{S}}_{q}^{5}=\frac{1}{|S_{q}^{5}|}\sum_{l\in S_{q}^{5}}{\hat{S}}_{l}\left(5\right)$$

for each subset $S_{q}^{5}=\left\{i\in \left\{1,\ldots ,N\right\}|{\tilde{p}}_{q-1}<p_{i}^{5}\leq {\tilde{p}}_{q}\right\},q=1,\ldots ,5$ (setting ${\tilde{p}}_{0}=1$) [36], and ${\hat{S}}_{KM,q}^{5}$ denotes the value of the Kaplan-Meier estimate of BCR-free survival ${\hat{S}}_{KM,q}\left(t\right)$ obtained for each subset $S_{q}^{5}$ and evaluated at *t* = 5 years. A second approach was used to assess model calibration for overall BCR-free survival estimation. This “overall survival” calibration curve was defined with respect to the quintiles $\left\{{\tilde{t}}_{1},\ldots ,{\tilde{t}}_{5}\right\}$ of the time-to-event data ${\left\{t_{j}\right\}}_{j=1}^{m}$ by coordinates $({\bar{S}}_{q},{\hat{S}}_{KM}\left({\tilde{t}}_{q}\right)),q=1,\ldots ,5$, where ${\bar{S}}_{q}$ denotes the sample mean survival likelihood

$${\bar{S}}_{q}=\frac{1}{|S_{q}|}\sum_{l\in S_{q}}{\hat{S}}_{l}\left(t_{l}\right)$$

in each subset $S_{q}=\left\{i\in \left\{1,\ldots ,N\right\}|{\tilde{t}}_{q-1}<t_{i}\leq {\tilde{t}}_{q}\right\},q=1,\ldots ,5$ (setting ${\tilde{t}}_{0}=0$) [36]. The confidence bands for each calibration curve were derived directly from the corresponding Kaplan-Meier estimates.

#### Predictive performance

Bootstrapped receiver operating characteristic (ROC) curves and corresponding area under the ROC curve (AUC) values were evaluated for all models to assess their predictive performance at specific, clinically relevant time points [37]. ROC is used to assess the correct classification of subjects as remaining BCR-free or not at a specific time-point. As certain points in time are considered clinically important for determination further treatment this analysis can give an indication of the performance of a model for this classification task. Decision curve analysis (DCA) [38] was carried out to provide complementary assessment in terms of the clinical value of the predictions from each model. DCA is also considered at specific time-points and again relates to the clinical importance of the models performance. The net benefit considered in the DCA is an indication of the increased or decreased number of patients who could see benefit in their treatment if the model was used for evaluation. Where a positive net benefit can be viewed as the proportion of patients who would benefit the from use of the model in a clinical decision making setting.

Lastly, feature selection rates from all model fits were recorded and analysed for all pipelines, to determine model stability in terms of their tendency to use the same features across all bootstrap resamples. This also allowed examination of what mRNA material was deemed relevant for BCR prediction.

## Results

Results from the quantitative analysis of the 16 modelling pipelines are presented hereafter with respect to each assessment criterion successively. For discrimination, the model outputs were compared by way of multiple pairwise comparisons using Wilcoxon signed-rank tests which are a nonparametric alternative to the t-test that allows for comparison of the centrality of the distributions [39]. 5% significance, after p-value adjustment for false discovery rate (FDR) was used as the level of significance [40]. These are the p-values discussed hereafter. For the predictive performance of the most discriminating pipelines, the AUC was compared by way of multiple pairwise comparisons using DeLong tests also at 5% significance, after p-value adjustment for FDR.

### Discrimination

Bootstrap C-indices and CIs for each model are shown in Table 1. The MSK model outperformed all other models using only clinical variables (*p* < 0.025). An important finding is that inclusion of mRNA variables significantly increased model discrimination performance for all modelling strategies (*p* < 0.001), compared to their baseline clinical counterpart and MSK. The gain obtained from inclusion of genetic information is observed in terms of the other performance aspects reported on hereafter.

**Table 1:** Bootstrap-corrected model discrimination performance in terms of C-index, and associated 95% bootstrap confidence intervals (CI). The best-performing pipeline for each modelling strategy is highlighted in bold.

Method (Pipeline)	Model	C-Index	CI
**Clinical Variables Only**	MSK	0.727	(0.644, 0.813)
	Cox	0.705	(0.631, 0.770)
	LASSO	0.708	(0.642, 0.760)
	Boosted	0.707	(0.629, 0.771)
	RSF	0.672	(0.606, 0.752)
**Clinical Variables and Correlation Prefiltered mRNA variables**	Cox	0.779	(0.440, 0.862)
	LASSO	0.669	(0.586, 9.753)
	Boosted	0.819	(0.748, 0.871)
	RSF	0.743	(0.677, 0.795)
**Clinical Variables and Univariate Cox Feature Selected mRNA variables**	**Cox**	**0.798**	**(0.710, 0.872)**
	**LASSO**	**0.819**	**(0.741, 0.892)**
	Boosted	0.798	(0.729, 0.859)
	RSF	0.767	(0.709, 0.826)
**Clinical Variables and Correlation Prefiltered and Cox Feature Selected mRNA variables**	Cox	0.788	(0.685, 0.853)
	LASSO	0.687	(0.500, 0.840)
	**Boosted**	**0.821**	**(0.748, 0.872)**
	**RSF**	**0.849**	**(0.770, 0.930)**

Table 1 further shows that pipelines using univariate Cox feature selection yielded significantly higher discrimination compared to those using correlation-based filtering only (*p* < 0.001), and combining both filtering strategies was optimal for boosted Cox and RSF models (*p* < 0.001). Overall, RSF achieved the highest discrimination performance, yielding statistically significant improvement over all other models (*p* < 0.001). All further analyses for each modelling strategy were carried out on their most discriminating pipelines.

### Calibration

**Fig 1:**
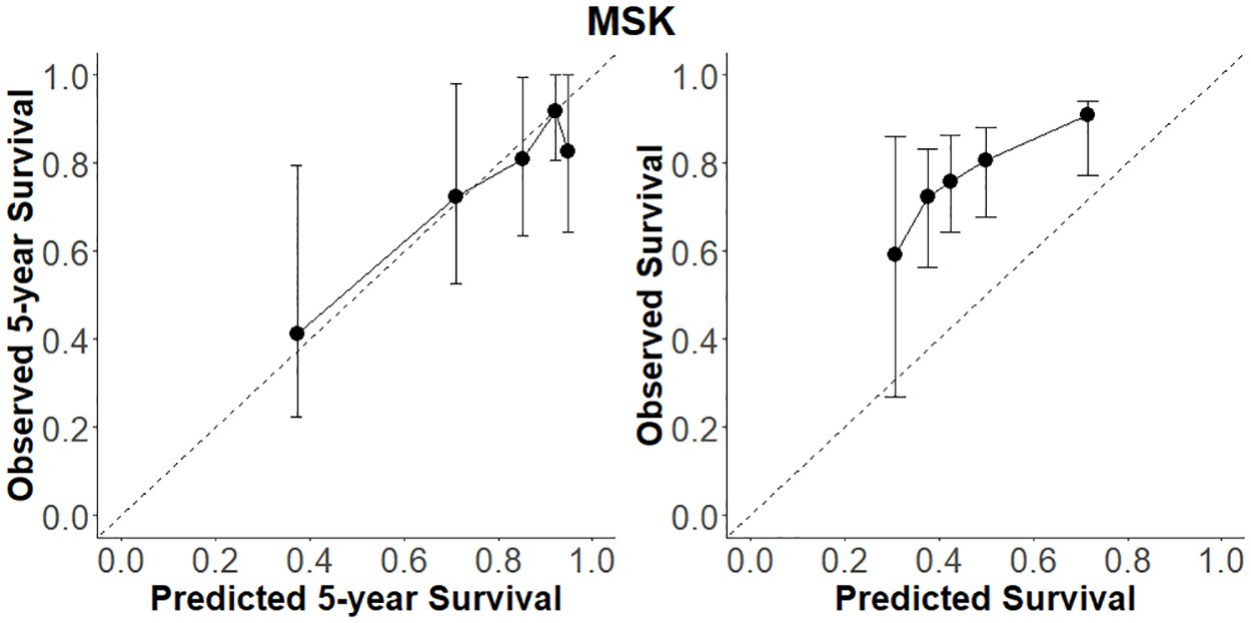
Calibration curves from MSK nomogram. Calibration curves for BCR-free survival estimation from the MSK nomogram at the 5-year horizon (left) and overall (right) with 95% confidence intervals.

**Fig 2:**
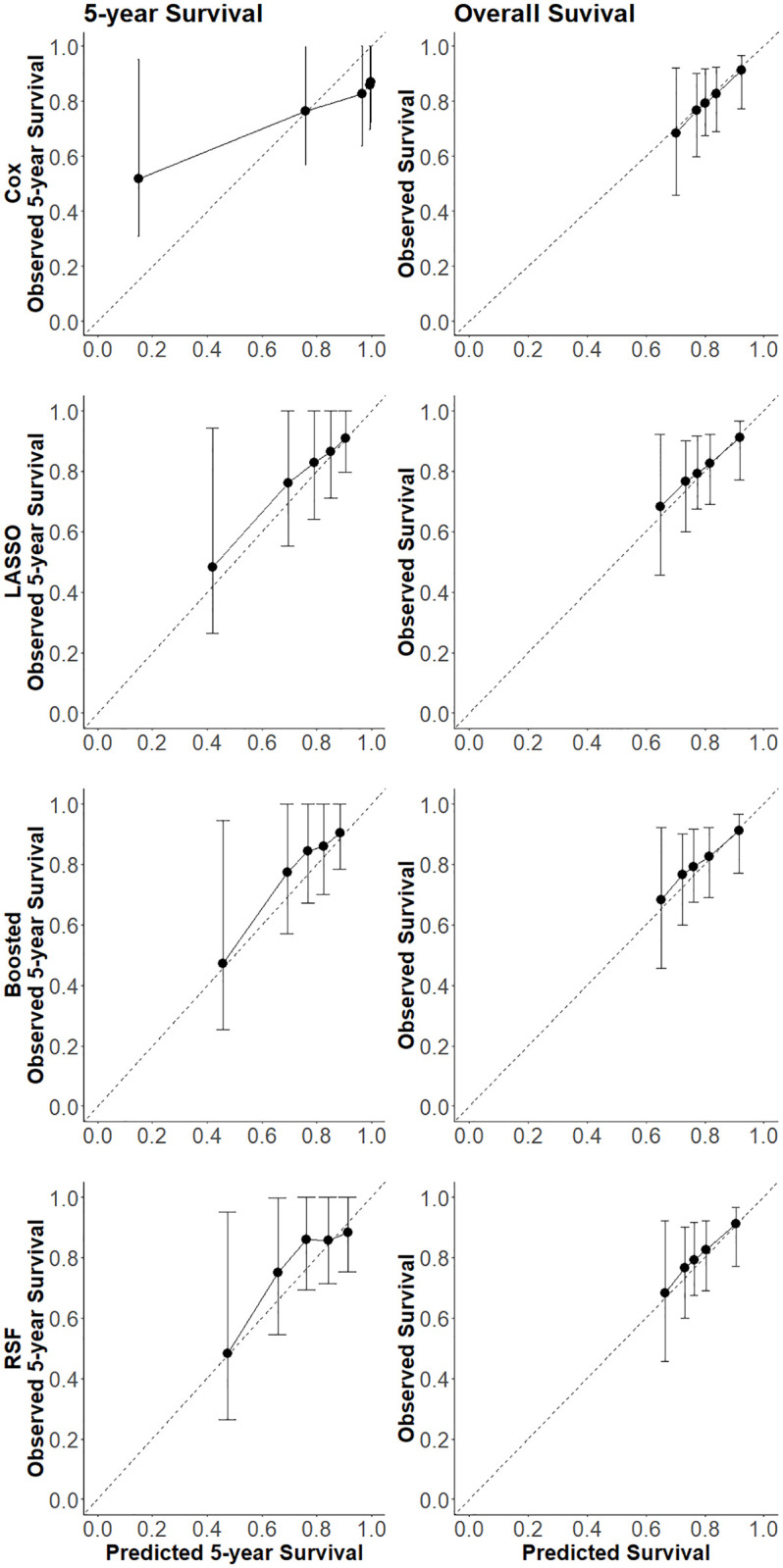
Model calibration curves. Model calibration curves at the 5-year horizon (left) and overall (right) BCR-free survival for the Cox, LASSO Cox, boosted Cox and RSF models from top to bottom, respectively with 95% confidence intervals.

The MSK nomogram reports on the probability of BCR-free survival at the 5-year horizon. All models were thus assessed for their calibration at 5 years and for overall BCR-free survival. Fig 1 shows calibration curves for the MSK nomogram. These indicate that the model performs adequately for 5-year survival, but severely underestimates the probability of overall BCR-free survival. Fig 2 illustrates how the most discriminating model pipelines from the Discrimination section yielded similar 5-year calibration compared to the MSK, except the Cox model, which yielded under-prediction and over-prediction at lower and higher survival probabilities, respectively. For overall survival, all models outperformed MSK with the Cox model having the best calibration.

### Predictive performance

**Fig 3:**
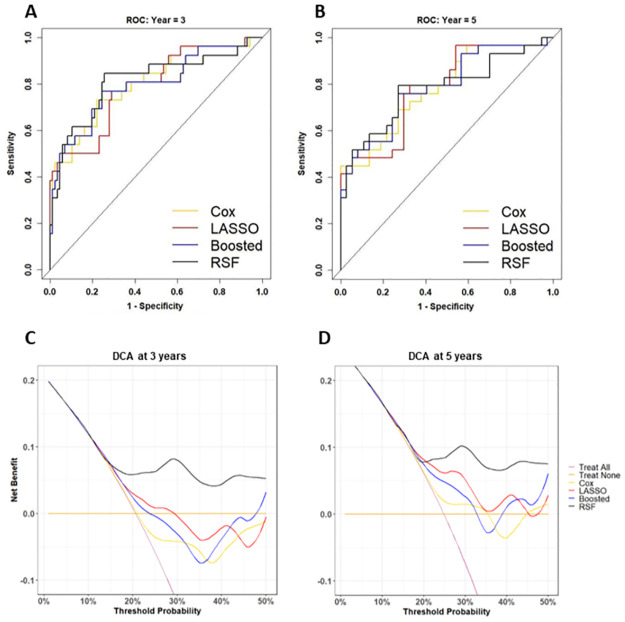
ROC and DCA. Model ROCs at the 3-year (A) and 5-year (B) BCR-free survival horizons for the models highlighted in bold in Table 1. Model DCAs at the 3-year (C) and 5-year (D) BCR-free survival horizons for the models highlighted in bold in Table 1.

ROC and DCA were carried out for 3-year and 5-year survival. The 3-year cut-off was chosen as it has been shown to be the time-frame with the highest recurrence rate [41], while the 5-year horizon was chosen in keeping with the current state-of-the-art cut-off [5]. ROC analysis output is shown in Fig 3 and Table 2. The highest OOB prediction was achieved by RSF (AUC = 0.812, CI = (0.702, 0.921)) at the 3-year horizon, and boosted Cox at the 5-year horizon (AUC = 0.786, CI = (0.672, 0.899)). Using the DeLong test for comparing AUC, there was no statistically significant difference in the methods at 3- or 5-year horizon. DCA output, shown in Fig 3, indicated that only the RSF model showed a continuous net benefit across all threshold probabilities for both the 3- and 5-year horizons. Although all models improved upon the “Treat All” method, no other methodologies showed benefit between the thresholds of 30% and 45% at 3 years. At 5 years, the LASSO Cox model retained some net benefit across most thresholds but was below that of the RSF model.

**Table 2:** OOB predictive performance in terms of AUC, and associated 95% bootstrap confidence intervals (CI). 3-year and 5-year endpoints for the best-performing pipeline for each modelling strategy.

Endpoint	Model	AUC	CI
**3-Years**	Cox	0.804	(0.700, 0.907)
	LASSO	0.789	(0.684, 0.895)
	Boosted	0.794	(0.684, 0.905)
	RSF	0.812	(0.702, 0.921)
**5-Years**	Cox	0.781	(0.668, 0.894)
	LASSO	0.782	(0.669, 0.895)
	Boosted	0.786	(0.672, 0.899)
	RSF	0.779	(0.660, 0.899)

### Model stability and feature selection

Stability of variable selection was assessed for all models that included mRNA variables. Table 3 summarises the most frequently selected variables for the top performing models. Overall, PSA level was the only variable consistently picked by all models, but some of the mRNA candidates were also selected frequently. Boosted Cox models yielded the highest selection rates for mRNA features, i.e. the most stable pipeline. The otherwise relatively low selection rates may indicate possible information overlap or similarities in interactions between different groupings of mRNA features.

**Table 3:** In-bag variable selection rates (%) of the top 10 most frequently selected variables in the models highlighted in Table 1. Variables explicitly linked to genetic information found in at least 3 of these 4 lists are highlighted in bold.

Cox		LASSO		Boosted		RSF	
**PSA**	**70**	**PSA**	**57**	**ESM1**	**75**	**PSA**	**97**
Clinical stage	35	**ESM1**	**46**	**DNAH8**	**54**	Gleason sum	83
Gleason sum	34	**DNAH8**	**40**	**PSA**	**49**	Clinical stage	62
**DNAH8**	**28**	PI15	36	**ABCC11**	**47**	**DNAH8**	**45**
**ABCC11**	**24**	Clinical stage	32	CD38	37	DUSP18	38
**ESM1**	**18**	**ABCC11**	**31**	EFCAB4B	35	ARMC4	32
PI15	18	Gleason sum	22	FZD5	31	NEU3	32
SOCS2	12	XBP1	22	ZHX3	29	GRM8	31
Ethnicity	11	PLA2R1	17	FAP	28	ERO1LB	29
HELB	11	SPAG1	16	SLC16A14	27	FAM122C	29

The RSF model consistently selected PSA (97%), aggregate biopsy Gleason score (83%) and clinical stage (62%) along with mRNA variables including DNAH8, which was also highly selected in the other top modelling approaches. The predictive ability of DNAH8 in PCa has been observed previously for assessing poor prognosis [42].

mRNA variables which occurred most frequently across the top models also included ESM1 and ABCC11. Although it has not been seen to predict for PCa survival previously, ABCC11 has shown predictive ability in recurrence of colorectal cancer [43]. Increased levels of ESM1 were previously linked to progression and development of metastasis [44]. Another mRNA variable selected in both the LASSO and Cox approaches was PI15 which has been identified as a biomarker for discrimination of metastatic progression [45].

PSA remained in the majority of the mRNA-including models, with above 49% selection rate for all the top models. Though it is considered a routine clinical variable as its testing is in the recommended guidelines [46], PSA is a protein produced from mRNA translation and can be considered a genetic biomarker itself [47]. Therefore, the most frequently used variables in our models for prediction of BCR were all genetic-based biomarkers.

**Fig 4:**
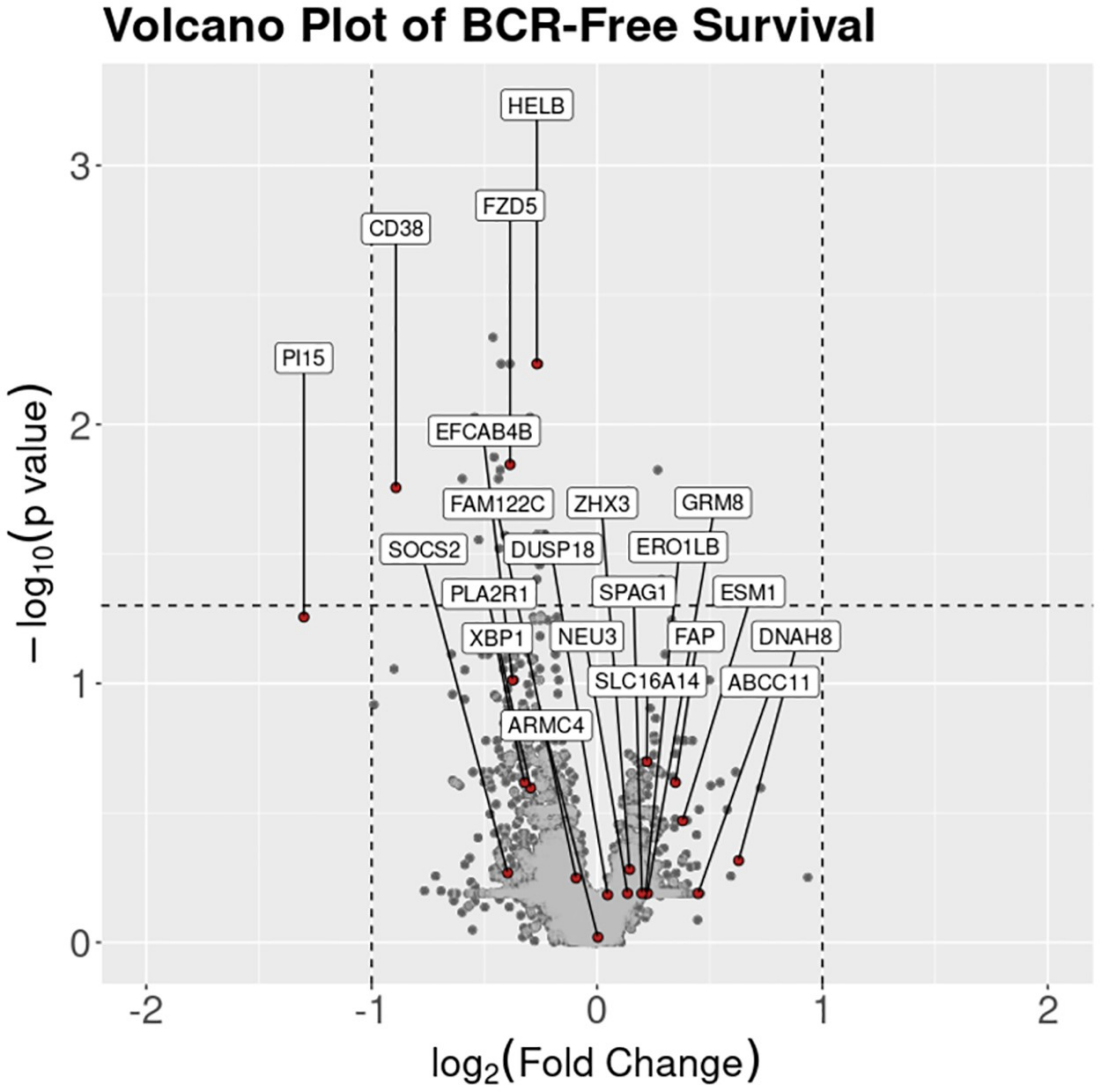
Volcano plot for BCR-free survival-related differentially expressed genes (dashed lines indicating fold change ≥ 2 and adjusted p-value < 0.05).

As this analysis was undertaken without prefiltering of the mRNA variables with prior clinical knowledge of their association with BCR-Free survival or PCa, post-hoc analysis was undertaken to inspect the most frequently selected variables. Fig 4 highlights the mRNA variables in the top ten most frequently selected variables in the most discriminative models. None of the mRNA variables meet the commonly implemented criteria for the determination of differentially expressed genes (DEG), with respect to a fold change ≥ 2 and adjusted p-value < 0.05 [48]. PI15 was the only variable with an absolute fold change greater than 2 and only three of the top selected variables (CD38, FZD5 and HELB) had an adjusted p-value less than 0.05. In order to investigate the potential clinical significance of the selected variables, they were tested for association with clinical variables; namely, aggregate biopsy Gleason score and clinical stage via one-way ANOVA. FAP, GRM8, ESM1, PLA2R1, EFCAN4B, FZD5, PI15, and CD38 where all statistically significant in biopsy Gleason score (adjusted p-value < 0.05). None of the mRNA variables where statistically significant in clinical stage.

## Discussion

Although none of the ML models outperformed the MSK nomogram when using only pre-operative clinical variables, all yielded significant performance improvement when including mRNA variables compared to their baseline clinical counterpart and the MSK model. The RSF yielding best discriminatory performance overall with good calibration as well as showing a continuous net benefit across all threshold probabilities. This demonstrates the potential of genetic information for prediction of BCR pre-operatively, and is one of our key findings. The ability of the RSF to capture nonlinear associations and complex interactions may explain some of the observed gain in performance over Cox-based models. The models where also compared to the post-operative models from our previous study [9], and it was found that the best performing model pipelines which included both clinical and mRNA variables also yielded significant performance improvement over the post-operative MSK nomogram and post-operative models using only clinical variables (*p* < 0.001). The best performing pre-operative RSF model was comparable in predictive performance to its post-operative counterpart as well as the post-operative boosted Cox model (*p* > 0.05) and showed statistically significant improvement over the best performing post-operative Cox and LASSO models (*p* < 0.001).

To the best of our knowledge, this study is the first to propose mRNA-based models for time-to-BCR prediction that are applicable pre-operatively. Incorporating mRNA measurements in pre-operative assessment would require an extraction from tumour-biopsy samples. As these samples are already required for routine clinical diagnosis the additional workload would relate solely to the mRNA analysis which has become more cost-reasonable and feasible in recent years. Another alternative would be to use a liquid-biopsy analysis of the blood-based measurements of these mRNA variables. This would however require substantial additional research into whether these variables are present in blood and if their levels are relatable to those in a tumour sample. ML methodologies are needed to leverage the predictive capabilities of mRNA data and thus a shift is needed from conventional nomograms. Improving their interpretability will be key in enabling clinical integration of such alternatives.

DNAH8, ABCC11 and ESM1 were among the list of mRNA variables with an important contribution to at least some of the models considered. These biomarkers were found in other studies to have a role in various forms of risk characterisation for prostate cancer and/or other diseases. DNAH8 and ESM1 both had selection rate ≥ 50% boosted Cox and RSF best-performing mRNA-inclusive pipelines in post-operative setting while ABCC11 had selection rate ≥ 35%. As the methodological approach implemented in this study did not undertake feature selection based on DEG investigation or association with clinical variables it was possible for previously uninvestigated variables to be selected and thus potentially new biomarkers for BCR-free survival prediction to be found. Though DNAH8 and ESM1 have previously been associated with prostate cancer they were not found to be BCR-free survival related DEGs in the post-hoc analysis and only ESM1 was found to be associated with a clinical variable (biopsy Gleason score). ABCC11 has not previously been found to have an association with prostate cancer and was also not a BCR-free survival related DEG, nor was in associated with the clinical variable in post-hoc analysis.

**Fig 5:**
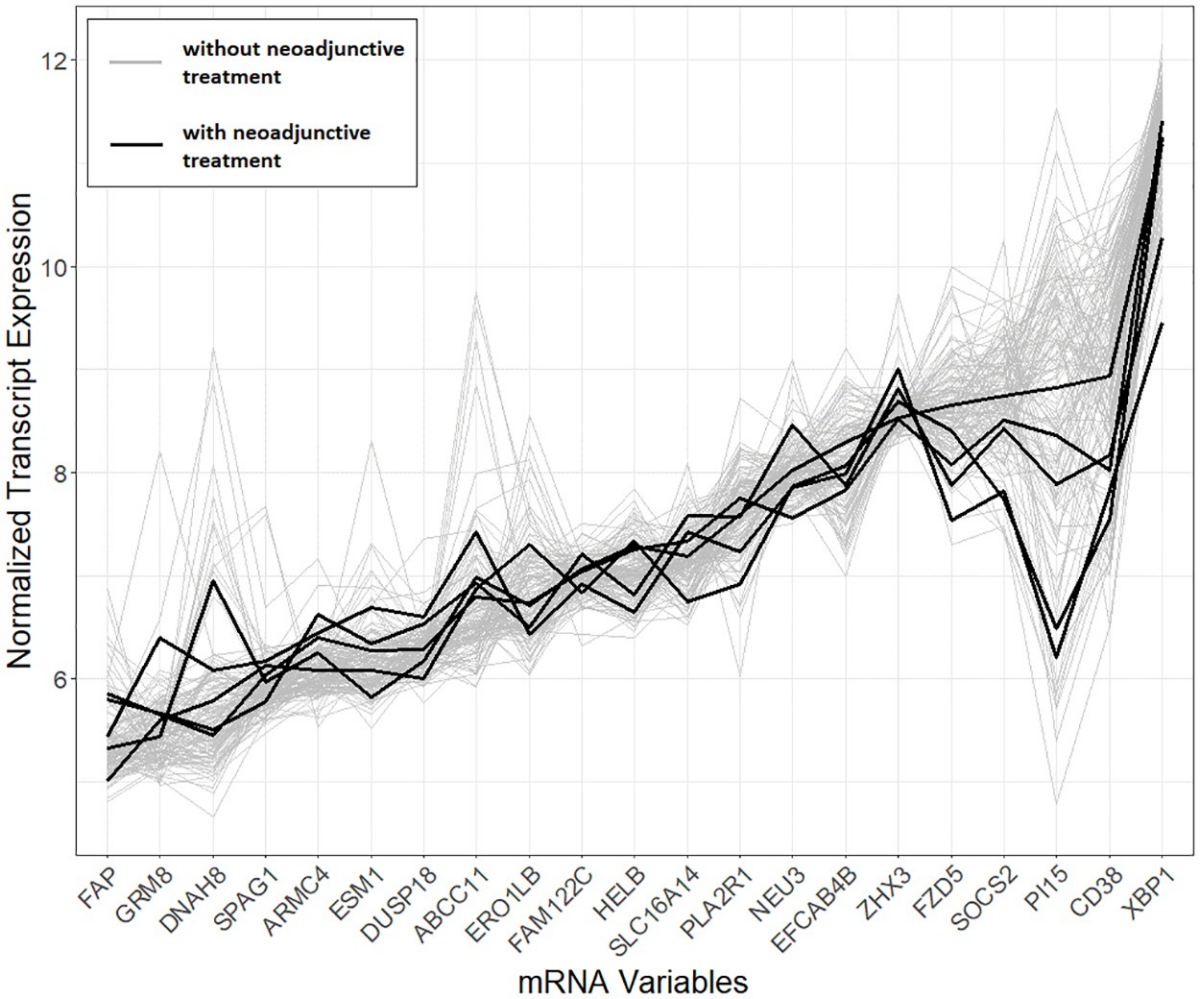
Normalised transcript expression levels. Normalised transcript expression levels for the most frequently selected mRNA variables (arranged with respect to increasing median value). Each line represents an individual patient’s expression with the patients receiving no neoadjunctive therapy in grey, and those who receive neoadjunctive hormone therapy in black. There is no evident departure from the grey pattern observed in the neoadjuvant hormone therapy signatures.

We aimed at assessing the potential for pre-operative BCR prediction, building upon the assumption that mRNA data used in the models is unlikely to have changed significantly between diagnosis and RP timepoints, as was observed in several studies on both PCa or other cancers [11–13]. An aspect that may have the potential to alter the mRNA expressions is neoadjunctive therapy. Of the 135 patients with mRNA information, five received neoadjunctive hormone therapy; however, on investigation, their mRNA expression for the variables most frequently selected by the models appeared in line with those of other patients (Fig 5). Validation of the findings on mRNA data acquired pre-operatively will be further explored in follow-on work.

The cohort size used in this study is relatively modest, and comes from a single centre. This limitation has the potential of bias which may reduce the generalisability of the model to more diverse datasets however it also alleviates other challenges typically found in multi-centre datasets, such as heterogeneity in demographic representation and discrepancies in clinical protocols that are not unusual in cohort sizes commonly available for this kind of study. External validation of the findings on a larger multi-centre dataset or multiple single-centred datasets including more varied populations will be key for future development and general clinical application. Specifically, making the findings more generalisable.

## Conclusion

This work demonstrated the potential of mRNA information (including in particular DNAH8, ABCC11, ESM1 and PI15) for improved pre-operative prediction of time-to-BCR in PCa, and is, to the best of our knowledge, the first to propose such models. Benchmarking of a number of machine learning methodologies that allow modelling of censored patient follow-up information indicated that RSF and boosted Cox models, in particular, were suitable candidates for the design of dedicated nomograms for this task. These implementations allowed leveraging relevant mRNA variables blindly from a large pool of features, without the reliance on previous findings about specific genetic biomarkers of interest. The pipelines that were implemented yielded promising performance on a relatively small cohort, in terms of model discrimination, calibration, and predictive performance of overall survival as well as 3- and 5-year survival, which are common current clinical endpoints. Following validation of these findings these models could be implemented into a clinical setting using biopsy samples or potentially liquid-biopsy with the appropriate regulatory requirements.
